# High power and low critical current density spin transfer torque nano-oscillators using MgO barriers with intermediate thickness

**DOI:** 10.1038/s41598-017-07762-z

**Published:** 2017-08-03

**Authors:** J. D. Costa, S. Serrano-Guisan, B. Lacoste, A. S. Jenkins, T. Böhnert, M. Tarequzzaman, J. Borme, F. L. Deepak, E. Paz, J. Ventura, R. Ferreira, P. P. Freitas

**Affiliations:** 1 International Iberian Nanotechnology Laboratory, INL, Av. Mestre José Veiga s/n, 4715-330 Braga, Portugal; 2 IN-IFIMUP, Rua do Campo Alegre, 687, 4169-007 Porto, Portugal

## Abstract

Reported steady-state microwave emission in magnetic tunnel junction (MTJ)-based spin transfer torque nano-oscillators (STNOs) relies mostly on very thin insulating barriers [resulting in a resistance × area product (*R* × *A*) of ~1 Ωμm^2^] that can sustain large current densities and thus trigger large orbit magnetic dynamics. Apart from the low *R* × *A* requirement, the role of the tunnel barrier in the dynamics has so far been largely overlooked, in comparison to the magnetic configuration of STNOs. In this report, STNOs with an in-plane magnetized homogeneous free layer configuration are used to probe the role of the tunnel barrier in the dynamics. In this type of STNOs, the RF modes are in the GHz region with integrated matched output powers (*P*_*out*_) in the range of 1–40 nW. Here, *P*_*out*_ values up to 200 nW are reported using thicker insulating barriers for junctions with *R* × *A* values ranging from 7.5 to 12.5 Ωμm^2^, without compromising the ability to trigger self-sustained oscillations and without any noticeable degradation of the signal linewidth (*Γ*). Furthermore, a decrease of two orders of magnitude in the critical current density for spin transfer torque induced dynamics (*J*_*STT*_) was observed, without any further change in the magnetic configuration.

## Introduction

The spin transfer torque (STT) effect^1–8^ allows the effective and selective manipulation of the magnetization of nano-magnets using local spin polarized electrical currents. It has been suggested as a key mechanism enabling a large number of spintronic devices, including magnetic random access memories (MRAM)^9^, domain wall based storage^10^ or spin transfer torque nano-oscillators (STNOs)^11–19^. With respect to STNOs, these devices take advantage of the STT effect to achieve RF emission from persistent magnetic precession, driven by DC currents. They show major advantages over conventional complementary metal-oxide-semiconductor (CMOS) and crystal based oscillators, such as being tunable by both electrical currents and magnetic fields, working in a large range of temperatures, having a broadband output and a nanometric footprint while keeping the compatibility with a CMOS backend process. STNOs are thus versatile and compact RF oscillators that can be vertically integrated with CMOS, making them highly attractive for applications such as chip-to-chip or wireless communications, microwave sources for nanosensors or phase-array transceivers^11, 20^.

There have been several proposals for alternative magnetic arrangements with different advantages explored in the context of specific applications. These include, homogenous in-plane oscillators^21^, vortex oscillators^16, 22^, oscillators integrating perpendicular polarizers^23^ or oscillators using free layers with canted magnetization^24, 25^ and point-contact nano-oscillators^26, 27^.

The STNOs with the largest reported integrated matched output power (*P*_*out*_) are fabricated starting from magnetic tunnel junction (MTJ) stacks based on CoFeB/MgO/CoFeB which benefit from their high tunnel magnetoresistance ratio (TMR)^11, 24, 25, 28, 29^. However, the MTJ endurance is limited by the dielectric breakdown of the MgO insulating layer. This is a critical point for STNOs wherein, to observe persistent oscillations, one must apply large and continuous current densities. This is a fundamental difference with respect to other applications exploring the STT phenomena, such as MRAM, which can use short pulses of very large current (and voltage) amplitude to excite the free layer magnetization. The barrier can sustain large pulsed voltages with amplitudes well above the DC breakdown value whilst large angle magnetization dynamics can still be excited. For STNO applications, the excitation current must be maintained in the steady state and therefore large current densities, capable of exciting persistent dynamics, must be reached with DC voltages that the MgO barrier can endure.

Despite the fact that a small number of reports achieved large *P*_*out*_ values with relatively thick MgO barriers [resistance × area product (*R* × *A*) > 4 Ωµm^2^]^23–25, 28^, most works still rely on very thin MgO barriers (*R* × *A* ~ 1 Ωµm^2^)^16, 20, 21, 30–34^. Hence, the consensus solution to excite STT excitations in nanofabricated MTJ stacks has been the use of ultra-low *R* × *A* barriers. In that respect, these ultra-thin MgO barriers can sustain high current densities but the so far unavoidable presence of a large density of defects and pinholes at such low barrier thicknesses (and low *R* × *A*) results in smaller TMR, lower breakdown voltage and an overall decrease in reliability and reproducibility^35^. Apart from the requirement of having ultra-low *R* × *A*, the role of the tunnel barrier on the properties of STNOs has been so far critically overlooked. The large majority of reported results rely in MTJs where the MTJ transport properties are dominated by defects. These defects are present already on the as-deposited state^29^ or, in some cases, are created prior to the dynamic characterization of the system by applying large currents that irreversibly change the transport properties of the device in a non-controlled way, which is required in order to observe STT persistent oscillations^36^. The effect of such defects on the dynamics is still not well accounted and, on top of that, the higher TMR and resistance of thicker MgO barriers should increase *P*_*out*_. The assessment of such large unexplored region for STNO operation could reveal crucial operating conditions to boost their applicability and offer new fundamental physical insights.

In in this work, the role of the tunnel barrier on the STNO dynamics is studied using homogeneous in-plane magnetized STNOs. Such STNOs have output powers in in the range of 1–40 nW and critical current densities required to excite auto-oscillations (*J*_*STT*_) are often larger than 10^6^ A/cm^2^ ^24, 28^. The STNOs were nanofabricated from MTJ stacks, deposited on 200 mm diameter wafers, and incorporating an MgO wedge (resulting in measured *R* × *A* values in the range 1–40 Ω µm^2^). Two wedge samples have been processed into nano-pillars with diameters of 200 nm which were characterized statically (TMR, low bias *R* × *A* and transfer curves, DC voltage/current breakdown) and dynamically (frequency spectrum versus bias current at fixed applied field). The results obtained in the two fabricated samples are consistent and clearly show that there is a trade-off between endurance to large currents (maximized for low *R* × *A* MTJs) and large TMR (maximized for large *R* × *A* MTJs), with an optimal *R* × *A* region showing the largest *P*_*out*_ in the 7.5–12.5 Ωµm^2^ range. In this region, STNOs with large impedance matched *P*_*out*_ values up to 200 nW are consistently found. This optimal *R* × *A* region was corroborated by micromagnetic simulations that revealed a good agreement with the experimental data. Furthermore, a decrease by 2 orders of magnitude of *J*_*STT*_ was observed when going from very thin (below 5 Ωµm^2^) to thicker MgO barriers. As a result, very small *J*_*STT*_ values (down to 1.17 × 10^5^ A/cm^2^) are achieved in the large *R* × *A* and large TMR region, resulting in an enhancement of the STNOs operational window. More precisely, as the *R* × *A* increases, the operational window [*J*_*STT*_ onset to breakdown current density (*J*_*break*_)] increases, contributing to an enhancement of the device robustness. Thus, the modification of the tunnel barrier thickness alone provides a mechanism to decrease the value of *J*_*STT*_, and simultaneously increasing *P*_*out*_, something of fundamental importance for all types of STNOs.

## Sample Nanofabrication

Two MTJ stacks incorporating MgO wedges were deposited over 200 mm Si 〈100〉 wafers in a Timaris Singulus tool, leading to a variable *R* × *A* over the wafer from below 1 Ωµm^2^ up to 40 Ωµm^2^ (corresponding to MgO thicknesses from ~0.6 to ~0.9 nm). The MgO barriers were deposited from MgO targets without subsequent oxidation. The two stacks deposited were S1: Substrate/100 Al_2_O_3_/5 Ta/50 CuN/10 Ru/50 CuN/20 Ru/17 Pt_38_Mn_62_/2 CoFe_30_/0.85 Ru/2.6 CoFe_40_B_20_/MgO wedge/2 CoFe_40_B_20_/10 Ru/150 Cu/30 Ru and S2: Substrate/100 Al_2_O_3_/3 Ta/30 CuN/5 Ta/17 Pt_38_Mn_62_/2 CoFe_30_/0.85 Ru/2.6 CoFe_40_B_20_/MgO wedge/1.4 CoFe_40_B_20_/10 Ru/150 Cu/30 Ru (thicknesses in nm). Despite other differences in the stack, the main variation between the two deposited wafers concern the free layer thickness: for S1, *t*_*CoFeB*_ = 2.0 nm and for S2, *t*_*CoFeB*_ = 1.4 nm. Both CoFeB layers have their magnetization in-plane, although in the case of S2 the CoFeB is already close to the transition between in-plane to out-of-plane magnetization (observed at ~1.1 nm of CoFeB). Most results reported here were collected from wafer S1, with S2 being mainly used to corroborate and demonstrate the reproducibility of the observed results. Upon deposition, the wafers were annealed for 2 h at 330 °C and cooled down under a magnetic field of 1 T along the easy axis defined during deposition.

**Figure 1:**
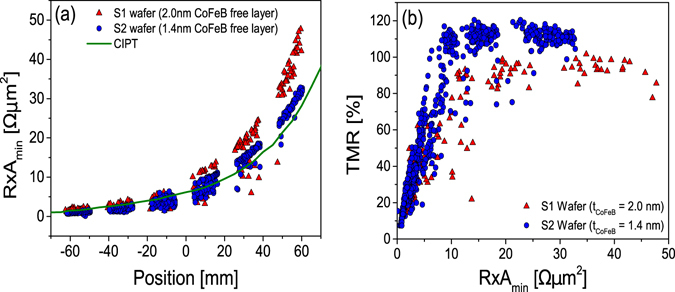
DC electrical characterization. *R* × *A* and TMR values extracted from the measured transfer curves in a 4-point contact geometry (red triangles correspond to S1 with *t*_*CoFeB*_ = 2.0 nm and the blue circles to S2 with *t*_*CoFeB*_ = 1.4 nm). (**a**) *R* × *A* of the measured MTJs and CIPT (of sample S1) measurements along the wafer position (green line) (**b**) TMR vs. *R* × *A* measured for the full collection of 200 nm MTJ pillars.

Both stacks were then patterned into circular devices with diameters of 200 nm. To that end, a nanofabrication method based on ion milling of the MTJ nano-pillars and an ion beam planarization step of an Al_2_O_3_ insulating layer was used. Each nano-pillar has four dedicated contact pads which were used to measure the TMR and *R* × *A* without any contribution of contact resistances. All the nano-pillars produced were measured under quasi-static magnetic field sweeps (up to 16 kA/m) in an automatic prober. Figure 1(a) shows the *R* × *A* values obtained from these transport measurements performed on the MTJ pillars along the MgO wedge position. The *R* × *A* values extracted from transport measurements in patterned nano-pillars follow the same trend observed in the current in-plane tester (CIPT) measurements. As the *R* × *A* decreases so does the dispersion of the measured *R* × *A*. This is attributed to the existence of intrinsic defects in the MgO layer. This interpretation is reinforced when the TMR values obtained from the patterned nano-pillars are plotted against the measured *R* × *A* value, as shown in Fig. 1(b). Below 10 Ωµm^2^ a strong linear correlation between TMR and *R* × *A*, which crosses the plot origin, is observed. This correlation is the signature of the presence of leakage currents through conduction channels that do not preserve the spin of the electrons. In nanometric sized nano-pillars this role is usually attributed to re-deposited material in the nano-pillar side-walls formed during the nano-pillar ion milling etching. In this case, however, an effort to monitor and remove the extra material in the nano-pillar sidewall was made during the nanofabrication process. The data in Fig. 1(b) indicates that this effort was successful: notice that the linear correlation between TMR and *R* × *A* exists only in the region bellow 10 Ωµm^2^. If re-deposited material were present in the nano-pillars produced, it would affect all nano-pillars, regardless of the *R* × *A* value. In fact, it should lead to a much larger TMR reduction in pillars with a large *R* × *A* compared to those with a small *R* × *A*. Still, a distribution of data points linking the high *R* × *A* data points to the plot origin is not observed. The conclusion is clear: the nanofabrication process was successful in preventing the formation of redeposited material shorting the tunnelling current through the MgO layer, but below the 10 Ωµm^2^ value, the thin MgO barrier contains intrinsic defects that partially de-polarize the current that crosses it^37^. As result, there is a cross-over *R* × *A* below which the TMR starts to decrease with respect to that obtained in thick barriers. Large TMR values can still be achieved in this region^21^, but the defects are still present and their effect in the electronic transport can be detected^37^. The TMR values achieved in sample S2 were larger than those achieved in sample S1. This is unexpected, since the free layer of sample S2 is thicker, and it is likely due to small variations in the nanofabrication process. Still, both samples show consistent trends.

## Characterization of the RF output in the frequency domain

To characterize the RF emission caused by STT excited oscillations of the free layer magnetization, a sub-set of the available MTJ nano-pillars was selected. The emission was studied at room temperature in the frequency window 3 Hz–10 GHz under a static bias current (*I*_*bias*_) and bias field (*H*_*bias*_) which were systematically swept within the limits of the experimental setup. As reported by other groups^12^, it was verified that *P*_*out*_ is maximized when applying a large static magnetic field in a direction close, but with a slight offset from the direction that sets the free layer in the anti-parallel direction. In the case of the results reported here, an external applied field *H*_*bias*_ = 16 kA/m applied along the plane (large enough to saturate the MTJ nano-pillars in the anti-parallel direction) was applied. The *I*_*bias*_ was then ramped up until STT persistent oscillations were observed in the frequency spectrum and then a small tilt of the magnetic field direction was introduced with the purpose of maximizing *P*_*out*_. Once the magnetic field direction was optimized, the systematic characterization of the output spectrum was performed, sweeping *I*_*bias*_ at constant *H*_*bias*_.

**Figure 2:**
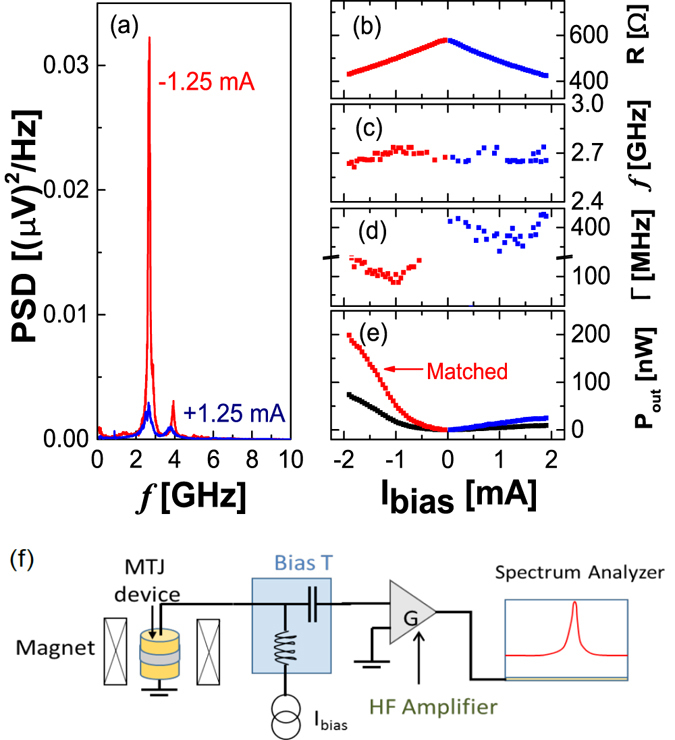
RF emission characterization. (**a**) Unmatched power spectral density measured at the amplifier input (PSD) with *I*_*bias*_ = ±1.25 mA. (**b**) Resistance, (**c**) frequency, (**d**) linewidth and (**e**) *P*_*out*_ as a function of *I*_*bias*_. The red (blue) points represent the integrated *P*_*out*_ matched to the load for negative (positive) *I*_*bias*_, while the black points represent the non-matched power. The magnetic field was kept constant (16 kA/m) in a direction close to the easy axis. (**f**) Schematic representation of the experimental setup used for the RF emission characterization.

An example of a frequency spectrum can be seen in Fig. 2(a). This result concerns a nano-pillar from wafer S1 with an *R* × *A* of 11.5 Ωµm^2^ and a TMR of 87.8% exhibiting a *P*_*out*_ of 200 nW at *I*_*bias*_ = −2 mA. As shown, the spectrum is highly asymmetric with respect to the bias current polarity. Large amplitude, small linewidth peaks consistent with STT enabled auto-oscillations are observed only for negative bias currents (negative current is defined here as electrons traveling from the pinned to the free layer). In such configuration, the STT destabilizes the anti-parallel configuration which is set by the magnetic field. Besides the large *P*_*out*_, the device also exhibits a reasonable linewidth (below 100 MHz). On the other hand, low power RF emissions with large linewidths consistent with thermal excitations are observed for positive currents, a configuration for which STT stabilizes the anti-parallel configuration. The small precession mode at 4 GHz might be caused by structural inhomogeneities or magnetic grains.

To compare the output power of nano-pillars with different *R* × *A*, care must be taken concerning the impedance mismatch in the acquired spectrum. The emission spectrum of the MTJ nano-pillars is amplified by an amplifier with a 50 Ω input impedance before being injected into the spectrum analyser where the spectrum is collected [Fig. 2(f)]. Due to the resistance mismatch between the amplifier input impedance (*R*_*L*_ ~ 50 Ω) and the MTJ, the measured output power is only a fraction of that actually emitted by the MTJ. The fraction of power amplified depends on the absolute resistance of the MTJ. For this reason, the output power collected in the amplifier for nano-pillars with different resistance values are not directly comparable. To account for the effect of the impedance mismatch the integrated matched output power *P*_*out*_ of each device was computed. To that end, for each measurement of the voltage [*V(f)*] at a certain *I*_*bias*_ and *H*_*bias*_ (and corresponding *R*), the integrated non-matched power (*P*_*measured*_) collected at the amplified input was calculated using:

1 $${P}_{measured}={\int }^{}\frac{V{(f)}^{2}-{V}_{0}{(f)}^{2}}{BW\cdot g(f)}\frac{1}{{R}_{L}}df$$

Here ${V}_{0}(f)$ is the voltage measured at ${I}_{bias}=0$, *BW* the measurement bandwidth (the value used in these measurements was 3 MHz) and *g*(*f*) the gain of the amplifier. The integrated matched output power *P*_*out*_ considering the measurement setup circuit is then calculated from^38^:

2 $${P}_{out}={P}_{measured}\cdot [\frac{{(R+{R}_{L})}^{2}}{4R\cdot {R}_{L}}].$$

The effect of this correction can be seen in Fig. 2(e). For a nano-pillar with a resistance between 400 Ω and 600 Ω (depending on *I*_*bias*_), *P*_*out*_ can be larger by a factor of 2 than *P*_*measured*_ at the amplifier input.

## Relation between TMR, *R* × *A* and *P*_*out*_

**Figure 3:**
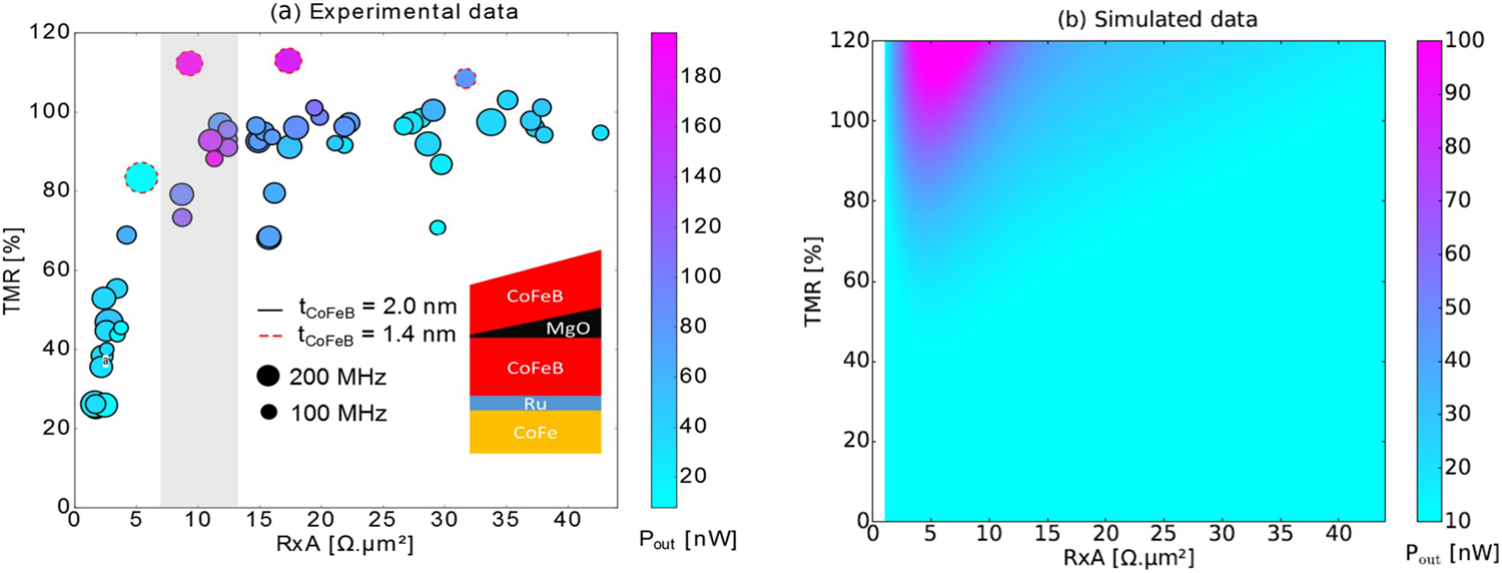
DC and RF electrical characterization. TMR versus *R* × *A* for all the studied STNOs (circles with black border correspond to S1 with *t*_*CoFeB*_ = 2.0 nm and the circles with dashed red border to S2 with *t*_*CoFeB*_ = 1.4 nm). The color scale of the points represents the maximum *P*_*out*_ of the RF emission and the size of the points the linewidth for the oscillation with the highest *Q*. The inset shows a schematic representation of the deposited MTJ stack. (**b**) Simulated *P*_*out*_ at the breakdown voltage, versus *R* × *A* and TMR (for *t*_*CoFeB*_ = 2.0 nm).

To clarify the role of the MgO thickness in the RF emission of the final devices, the RF emission of a sub-set of the devices represented in Fig. 1 was characterized. Figure 3(a) shows the position of the selected devices in the TMR vs. *R* × *A* phase space together with the maximum measured *P*_*out*_ value which is represented as a colour scale. The size of each dot encodes the linewidth information at the *I*_*bias*_ value that maximizes the quality factor (*Q* = *P*_*out*_ */Γ*) with larger dots representing larger linewidths. It is important to note that the maximum *P*_*out*_ represented in Fig. 3(a) were obtained under different *I*_*bias*_ values for different devices. Due to the different impedance values of the patterned nano-pillars (which have all the same area, but different MgO thicknesses) the optimum current that maximizes *P*_*out*_ depends on the position of the devices on the wafer.

It is clear from Fig. 3(a) that *P*_*out*_ is maximized precisely in the *R* × *A* region between 7.5–12.5 Ωµm^2^. In this region, *P*_*out*_ is larger by a factor of 5 when compared to that obtained in the ultra-low *R* × *A* region close to 1 Ωµm^2^ which is usually targeted in STNO devices. At a first look, one could argue that the optimal *R* × *A* region that maximizes *P*_*out*_ is just the region that displays higher TMR with the lowest possible *R* × *A*. However, if we compare the STNO with the highest TMR in the region below 5 Ωµm^2^ (69%) with the oscillator with the lowest TMR in the optimal region (73%) the *P*_*out*_ values are still higher in the optimal region (65 and 121 nW, respectively). Furthermore, even though there is some variability in the *P*_*out*_ of oscillators with similar positions in the TMR vs *R* × *A* plot, the lowest measured *P*_*out*_ value in the optimal region is always larger than the highest *P*_*out*_ for the small *R* × *A* samples (below 5 Ωµm^2^). Therefore, even though lower *R* × *A* values allow the use of higher currents that may excite larger oscillations, the decrease of *R* × *A* is not always the best option to achieve optimal STNOs. In fact, the higher resistance characteristic of a thicker MgO results in a larger voltage variation of the oscillator even for similar TMR ratios. A similar conclusion can be extracted if the *P*_*out*_ for STNOs in the optimum *R* × *A* region is compared with that of devices in the larger *R* × *A* range. This occurs because for more resistive MTJs, *J*_*break*_ is achieved before the excitation of large magnetic precessions, leading to an optimal *R* × *A* region. The observed variation of the *P*_*ou*t_ values likely results from the unavoidable process variability (small differences in sizes, density of defects or edge roughness). Despite this factor, the large number of characterized devices allowed us to determine clear trends as a function of *R* × *A*. This behaviour was observed and reproduced in the two wedge wafers nanofabricated, despite the different magnetic configurations (*t*_*CoFeB*_ = 2.0 nm for S1 *t*_*CoFeB*_ = 1.4 nm for S2).

To further corroborate the observation of an optimal *R* × *A* region where *P*_*out*_ is maximized, these experimental results were compared with micromagnetic simulations of the STT-induced dynamics. The simulations were performed using the object oriented micromagnetic framework (OOMMF)^39^ to solve the Landau-Lifshitz-Gilbert-Slonczewski (LLGS) equation^1^. A 2.0 nm thick CoFeB free layer patterned in a 200 nm diameter pillar was considered (matching the stack and geometry of S1 devices). Apart from the geometry, the remaining parameters that were kept constant in the simulations were: Gilbert damping α = 0.01, free layer magnetization M_s_ = 1.36 × 10^6^ A/m and applied field *H*_*bias*_ = 16 kA/m. The interfacial perpendicular magnetic anisotropy induced by the MgO layer was also considered (interfacial anisotropy constant of 1.5 × 10^−3^ J/m^2^ extracted from magnetic measurements).

The simulations were performed with a bias current density of $\,{J}_{bias}={V}_{break}/(R\times A)$, that were measured experimentally as a function of *R* × *A* using a ramp procedure (in a sub-set of devices of Fig. 1). The applied current was successively increased until dielectric breakdown is observed and the measurements fitted to an exponential law. This is the maximum possible current density that can be reached and, theoretically, the current that maximizes *P*_*out*_ (within the range of the experimentally achievable values). On the other hand, the spin current polarization (*P*) was determined using the TMR value and Jullière’s model^40^ $[TMR=\,2{P}^{2}/(1-{P}^{2})$]. Hence, the STT-induced dynamics could be computed using the TMR and *R* × *A* as input parameters which were swept systematically within the limits of Fig. 3(a).

Then, the *P*_*out*_ delivered to a load with impedance *R*_*L*_ was then calculated from the induced dynamics using^11, 41^:

3 $$\langle {P}_{out}\rangle \approx \frac{\langle {V}_{out}^{2}\rangle }{{R}_{L}}=\frac{{{I}_{bias}}^{2}\Delta {R}^{2}{R}_{L}}{2{(R+{R}_{L})}^{2}}.$$

Here, ${V}_{out}(t)={\rm{\Delta }}R\cdot \,\cos (wt){I}_{bias}$ is the RF output voltage, *R* stands for resistance of the STNO and *ΔR* stands for the peak-to-peak amplitude of the STNO impedance variation during an oscillation period.

Therefore, the input *R* × *A* value will set the *I*_*bias*_ used, along with the value of *R* and *ΔR* (this last parameter also depends on the TMR). As for the input TMR value, it has a twofold influence on *P*_*out*_. On one hand, *ΔR* for a given oscillation amplitude is proportional to the TMR value. On the other hand, the oscillation amplitude for a given current density depends on the spin current polarization which is also linked to the TMR value.

During the simulations, the *P*_*out*_ values delivered to a matched load were computed from equation (3) by setting *R*_*L*_ = *R*, with the result of this procedure being plotted in Fig. 3(b). Remarkably, the optimal *R* × *A* region where *P*_*out*_ is maximized was also observed in the simulated case, although with the optimal region occurring for smaller *R* × *A* values (maximum around 8 Ωµm^2^). The calculated *P*_*out*_ values (up to 100 nW) are also lower than the ones that were experimentally measured (up to 200 nW). A part of this discrepancy can be attributed to the thermally-induced precessions that were neglected in the simulations. Note that an overall increase of the magnetization oscillation amplitude would increase *P*_*out*_ more significantly for larger *R* × *A* values, moving the optimal *R* × *A* region to values closer to the experimental data. Nevertheless, the simulations corroborate the observation of an *R* × *A* region that maximizes *P*_*out*_.

## Critical current densities for STT

To achieve very large amplitude oscillations before the dielectric breakdown of the MTJ, a very low critical current density for STT-induced magnetic precession *J*_*STT*_ is required. In fact, it was shown theoretically that *P*_*out*_ increases with *J/J*_*STT*_^42^. The fact that STT excited oscillations are observed in the full measured *R* × *A* range is surprising. Since these oscillations can only be obtained when the condition $\,{J}_{STT} < {J}_{break}$ is met, the observation of oscillations even for *R* × *A* values of ~40 Ωµm^2^ can only be understood if, in this large *R* × *A* range, *J*_*STT*_ is unexpectedly small or *J*_*break*_ is unexpectedly large.

**Figure 4:**
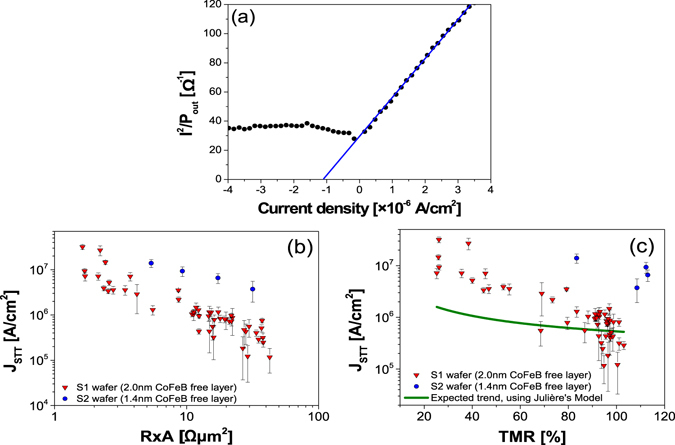
Critical current density for STT-induced oscillations. (**a**) Example of the determination of the critical current by the x-axis interception of the linear fit of *I* *²* */* *P* in the thermally activated region. Calculated values of *J*_*STT*_ as a function of (**b**) *R* × *A* and (c) TMR for *t*_*CoFeB*_ = 2.0 nm (red triangles) and *t*_*CoFeB*_ = 1.4 nm (blue circles).

The values of *J*_*STT*_ can be calculated by identifying the deviation from the linear dependence of the inverse power on the bias current. An example of this derivation is plotted in Fig. 4(a), where the value of the critical current density *J*_*STT*_ was extracted by extrapolating the inverse power times the current squared (${{I}_{bias}}^{2}/{P}_{out}\to 0$). *J*_*STT*_ can be derived using the relation $({{J}_{bias}}^{2}/{P}_{out})\propto ({J}_{STT}-{J}_{bias})$, valid in the thermally activated region and noting that, as the inverse power approaches zero, ${J}_{bias}\to {J}_{STT}\,$ ^24, 42^. Thus, *J*_*STT*_ is determined by the x-axis intercept with the linear fit in the thermally excited region. In Fig. 4(b) and (c) the calculated values of *J*_*STT*_ are shown as a function of *R* × *A* and TMR, respectively. From the *R* × *A* dependence of S1 [Fig. 4(b); red triangles] one can observe that *J*_*STT*_ sharply decreases by 2 orders of magnitude as the MgO thickness is increased. In fact, the TMR dependence [Fig. 4(c)] indicates that large TMR values, characteristic of continuous MgO barriers, depict significantly lower values of *J*_*STT*_ with smaller error bars (note the logarithmic scale). The values of *J*_*STT*_ obtained for S2 (blue circles) corroborate the observed tendencies but with larger values of *J*_*STT*_. Further work is still required to understand the reason of this discrepancy. Nevertheless, despite the sample-to-sample variation, the observed *J*_*STT*_ dependence (as a function of *R* × *A* and TMR) is consistent in both samples. This result unveils the possibility to decrease the value of *J*_*STT*_ just by using thicker MgO barriers.

In the macrospin approximation, the critical current density for STT oscillations (*J*_*STT*_) can be written as^43^:

4 $${J}_{STT}=\frac{2e{\mu }_{0}{M}_{s}\alpha d}{\hslash }(\frac{{M}_{eff}}{2}+{H}_{bias})\cdot \frac{1}{P}$$

where *e* is the charge of the electron, ${\mu }_{0}$ is the permeability of free space, *ħ* the Planck constant, *M*_*s*_ the magnetization saturation, *α* the Gilbert damping constant, *d* the thickness of the free layer, *M*_*eff*_ the effective demagnetizing field (given by the demagnetizing factor minus the interface perpendicular magnetic anisotropy) and *P* the spin polarization.

The value of *J*_*STT*_ is expected to be inversely proportional to the spin polarization of the current. Jullière’s model was used to correlate the TMR with the spin polarization and, in conjugation with equation (4), the expected trend of *J*_*STT*_ as a function of TMR was estimated. This was performed, using the value of *J*_*STT*_ measured for S1, with the calculated trend represented by the green line in Fig. 4(c). Even though the predicted trend indeed reveals a decrease of *J*_*STT*_ with the TMR, the experimentally observed dependence is not completely consistent with the model. The discrepancy between the model and the experimental data can be attributed to different effects not accounted for in this simple model. As a first possibility, the TMR value used to perform the fit was measured at low bias and decreases for higher bias voltages. The TMR at *J*_*STT*_ could not be determined since the RF characterization was performed with 2-point contacts (associated contact resistance) and the measurements were destructive (performed until MTJ breakdown). It is expected that in conditions consistent with STT excitations the spin polarization of the current being injected in the free layer decreases compared to a low bias condition. However, this decrease in the spin polarization under STT conditions should be more pronounced for devices in the large *R* × *A* range when compared to devices in the low *R* × *A* range (which have lower breakdown voltages and therefore a smaller TMR decrease with increasing *I*_*bias*_). So, this effect should have a contribution which opposes the trend observed experimentally. Either this effect has a small contribution or there are some other factors playing a more important role. A second possibility consists on the ferromagnetic coupling (*H*_*F*_) which depends on the MgO thickness due to the orange peel coupling^44^. The *H*_*F*_ field is larger for thinner barriers, and it can easily reach values of the order of 8 kA/m near the 1 Ωµm^2^ range, which is comparable to the applied field *H*_*bias*_ = 16 kA/m, meaning that the effective field acting on the free layer can have a non-negligible dependence on *R* × *A* as well. However, this effect is expected to be small since *H*_*F*_ is around one order of magnitude smaller than the effective demagnetizing field. Finally, as discussed previously, the data of Fig. 1 indicates that for devices with *R* × *A* < 10 Ωµm^2^ there are intrinsic defects in the MgO barrier which provide alternative conduction channels dominated by transport mechanisms other than tunneling^37^. Only the fraction of current which crosses the MgO barrier through tunnelling is described by Jullière’s model. In the presence of such defects, the fraction that is not described by this model and does not conserve the spin of the electrons increases as *R* × *A* decreases. This is perceived as a decrease in the spin polarization which results in larger *J*_*STT*_ values compared to a scenario where spin conservative tunnelling is the only transport mechanism. This effect is consistent with the mismatch observed in Fig. 4(c), although a better understanding of the different transport mechanisms and the fraction of current carried by each of them is required for a quantitative analysis.

## Operational window

**Figure 5:**
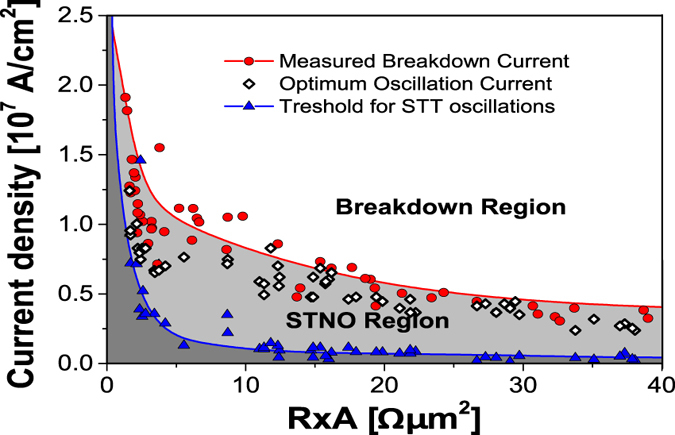
Range of operation of STNOs. Critical current density for STT-induced oscillations *J*_*STT*_ (blue triangles), breakdown current density *J*_*break*_ (red circles) and current for which the highest *Q* is achieved (white diamonds). The lines are splines fitted to the data separating the region without STT effects (dark grey), the STNO region (light grey) and the breakdown region (white). The considered sample was S1 with *t*_*CoFeB*_ = 2.0 nm.

For STNOs to reach commercial applications it is important to have a large *P*_*out*_ and a small bandwidth but also stable devices that achieve large oscillations for safe conditions with currents well below breakdown. To determine this range of operation, in Fig. 5 it is depicted the current density values at which the *Q* factor (*P*_*out*_ */Γ*) is maximized (white diamonds) along with *J*_*break*_ (red circles) and *J*_*STT*_ (blue triangles) for each STNO characterized in wafer S1.

For low *R* × *A* (below 5 Ωµm^2^) the current density that optimizes *Q* and the *J*_*break*_ values are quite large. However, the *J*_*STT*_ values are also significantly large so that the region for STNO operation (STNO region) is particularly thin. The reason for this is that despite the large *J*_*break*_, the breakdown voltage (*V*_*break*_) is smaller in this region. In fact, while for *R* × *A* values below 5 Ωµm^2^ one has *V*_*break*_ ~ 0.35 V (due to the presence of small defects in the insulating layer), above 5 Ωµm^2^, *V*_*break*_ increases to ~1 V. As *R* × *A* increases, the MgO barrier gets thicker and its quality improves (more continuous and better defined crystalline texture), the voltage endurance of the MTJ is higher and simultaneously the values of *J*_*STT*_ get significantly lower. Thus, although the maximum current density endured by the tunnel barriers decreases with *R* × *A*, the value of *J*_*STT*_ also decreases but at an even faster rate which results in a broader STNO operating region.

Moreover, the higher resistance of these MTJs gives rise to larger voltage variations for the same magnetic precession amplitude leading to larger *P*_*out*_ values observed in the intermediate *R* × *A* region. When *R* × *A* further increases, *J*_*break*_ steadily decreases. For high *R* × *A* the currents that maximize *Q* are close to (or even coincide) with the breakdown, while for lower *R* × *A* values, they are closer to the bottom limit of the STNO region. This means that, for *R* × *A* values above ~20 Ωµm^2^, only small amplitude oscillations can be achieved before breakdown occurs. This leads to a new decrease of *P*_*out*_ and confirms the optimal *R* × *A* region between 7.5 and 12.5 Ωµm^2^.

## Open prospects

Despite the large effort of the STNOs community to work in the lowest possible *R* × *A* range, this work presents a large set of consistent data showing that thicker MgO barriers increase the *P*_*out*_ of these oscillators. From an application point of view this is a twofold advantage since *P*_*out*_ is increased and thicker and more homogeneous MgO barriers have less defects and higher reproducibility. This optimal *R* × *A* region is situated within 7.5–12.5 Ωµm^2^ where *P*_*out*_ values up to 200 nW were observed, which are a factor of 5 larger than those obtained from devices with *R* × *A* ~ 1 Ωµm^2^. The results were corroborated by micromagnetic simulations and by a second fabricated MTJ incorporating an MgO wedge, both depicting the optimal region for maximized *P*_*out*_. The main fact responsible for this large output is the low *J*_*STT*_ (down to 1.17 × 10^5^ A/cm^2^) obtained for the more continuous and crystalline MgO. Further investigation is still required to fully understand the mechanisms responsible for the low values of *J*_*STT*_. Another worthwhile aspect of the results shown here is that the intermediate thickness of the MgO barrier can be applied to different STNOs geometries. The increase of the MgO thickness had no other discernible impact in the main operational figures of merit of the STNOs apart from the increase of *P*_*out*_ (frequency of operation and linewidth remained unchanged). It is therefore expected that the reported *P*_*out*_ increase in the intermediate MgO thickness range reported here could have a cumulative effect when combined with other improvements of magnetic nature (such as vortex oscillators, STNOs incorporating perpendicular magnetic anisotropy or perpendicular polarizers) or device configurations (synchronized oscillators) that are also known to result in an increase of *P*_*out*_ when compared to homogeneous in-plane magnetization STNOs.

## Methods

### Nanofabrication process

The MTJ nano-pillars were patterned in a 3-step ion milling process which is monitored in real time by a secondary ion mass spectrometer (SIMS). During the 1^st^ step, a capping layer is patterned (150 nm Cu/30 nm Ru), using high etch rate conditions. During the 2^nd^ step the magnetic layers and MgO tunnel barrier are patterned using low etch rate and low energy conditions (with an incoming ion energy of 150 eV) that minimize the amount of damage created in the nano-pillar by ion bombardment. This step is stopped between 10–50 nm (depending on the stack) below the bottom layer of the antiferromagnetic layer, ensuring that all the ferromagnetic layers are confined within the nano-pillar shape. During the 3^rd^. step a grazing angle milling is used to clean the redeposited material from the nano-pillar sidewalls. The nano-pillar shape and amount of redeposited material are evaluated using HR-SEM conducted between steps 2 and 3. After a first side-wall cleaning step, new HR-SEM images of the nano-pillars are collected and if necessary the sidewall cleaning step is extended. This cycle is repeated until the HR-SEM images reveal nano-pillar images free of any noticeable extra material. Upon defining the nano-pillar, an 800 nm Al_2_O_3_ insulating layer is deposited. The electrical contact to the top part of the nano-pillar is established by an ion milling planarization process where the oxide layer is bombarded, planarized and thinned down until the buried sacrificial contact layer is exposed. The stop point for this process is established by monitoring the evolution of the oxide topography on top of the nano-pillar using HR-SEM.
